# Durability against Wetting-Drying Cycles of Sustainable Biopolymer-Treated Soil

**DOI:** 10.3390/polym14194247

**Published:** 2022-10-10

**Authors:** Antonio Soldo, Marta Miletic

**Affiliations:** 1 Department of Civil and Environmental Engineering, Auburn University, Auburn, AL 36849, USA; 2 Department of Civil, Construction and Environmental Engineering, San Diego State University, San Diego, CA 92182, USA

**Keywords:** biopolymer-treated soil, Xanthan Gum, Guar Gum, soil strength, durability, cyclic wetting-drying

## Abstract

The world today is more oriented towards sustainable and environmental-friendly solutions in every field of science, technology, and engineering. Therefore, novel sustainable and eco-friendly approaches for soil improvement have also emerged. One of the effective, promising, and green solutions is the utilization of biopolymers. However, even though the biopolymers proved to be effective in enhancing the soil-mechanical properties, it is still unknown how they behave under real environmental conditions, such as fluctuating temperatures, moisture, plants, microorganisms, to name a few. The main research aim is to investigate the durability of biopolymer-improved soil on the cyclic processes of wetting and drying. Two types of biopolymers (Xanthan Gum and Guar Gum), and two types of soils (clean sand and silty sand) were investigated in this study. The results indicated that some biopolymer-amended specimens kept more than 70% of their original mass during wetting-drying cycles. During the compressive strength analysis, some biopolymer-treated specimens kept up to 45% of their initial strength during seven wetting-drying cycles. Furthermore, this study showed that certain damaged soil-biopolymer bonds could be restored with proper treatment. Repeating the process of wetting and drying can reactivate the bonding properties of biopolymers, which amends the broken bonds in soil. The regenerative property of biopolymers is an important feature that should not be neglected. It gives a clearer picture of the biopolymer utilization and makes it a good option for rapid temporary construction or long-standing construction in the areas with an arid climate.

## 1. Introduction

The expansion of cities often causes the need to construct in an unfavorable environment and on soils with undesirable mechanical characteristics. As a solution, soil’s engineering properties can be improved by adding different chemical additives. Currently, cement is one of the most commonly used additives. However, the use of cement raises a series of environmental problems from which the contribution to CO_2_ concentration on the planet is the most concerning. From the data in 2016, the production of cement contributes approximately 7.4% to the world’s CO_2_ emissions [1]. Furthermore, the use of cement can irreversibly affect the urban environment. Increased urban water runoff, vegetation growth prevention, and heat islands are some of the side effects of using cement as soil stabilizer [2]. Therefore, the need for a sustainable, green, and effective solution for enhancing soil characteristics is continuously increasing.

New bio-inspired solutions for the improvement of mechanical characteristics of soil, such as biopolymer-soil mixtures, are proved to be quite effective [2,3,4,5,6]. A biopolymer is a chain of smaller molecular units extracted from nature-made materials, such as wood, vegetable, algae, and animal shells. To the best of the authors’ knowledge, no negative effect of biopolymers on the environment has been reported. Throughout recent history, biopolymers were used in the food industry, the cosmetic industry, medicine, and agriculture [7,8,9,10,11]. In previous research, it was found that biopolymers, such as xanthan gum, guar gum, beta-glucan, and chitosan, can improve the strength of soil [5,6,12,13,14,15]. In addition, some biopolymers proved effective in reducing the collapsibility of soil [16] and erosion [17,18,19]. 

However, the main concern is the durability of biopolymer-amended soils while being exposed to environmental conditions such as wind, moisture, and temperature fluctuations. Kavazanjian et al. [18] investigated the effect of wind on erosion properties of the biopolymer-amended soil. Biopolymer emulsion was sprayed on the surface of the soil, and wind flow was blown over the soil surface. The major finding was that biopolymers could reduce wind-induced detachment of soil particles, but that ultraviolet radiation and heat can diminish their effect. 

To date, the research on the durability of biopolymer-amended sand remains limited and insufficiently investigated. Chang et al. [20] explored the properties of biopolymer-amended sand against cyclic wetting-drying. They performed a series of unconfined compression tests on gellan gum-improved sand specimens after each wetting and drying cycle. Chen et al. [21] performed a series of direct shear tests on xanthan gum-improved sand. Both of the above-mentioned research studies have found that the strength of biopolymer-amended sand ultimately decreases due to cyclic wetting and drying. Some limitations of each of the mentioned studies are that one type of testing was conducted and the soil (sand) was amended with only one type of biopolymer. Some additional research in the field of cyclic wetting-drying of biopolymer-treated soil is presented in Table 1.

The main research aim of our study is to investigate the durability of biopolymer-improved soil on the cyclic processes of wetting and drying. Two soil types were investigated in this study, clean sand and silty sand. Additional testing variables were biopolymer type and concentration. In particular, the soil was treated with two types of biopolymer (Xanthan Gum and Guar Gum) at three different biopolymer concentrations (0.5%, 1%, 2%). Furthermore, plain and biopolymer-amended specimens were tested under two types of water-durability tests. Considering that Xanthan Gum and Guar Gum emerged as biopolymers with high potential for soil stabilization, the investigation of their durability will have a significant impact on their utilization in civil engineering practice. 

## 2. Materials and Methodology 

### 2.1. Base Soil 

To investigate the effect of the soil type on the biopolymer-amended soil durability, two types of soil were investigated in this study: silty sand (SM), and poorly graded sand (SP). The soils were classified according to the following standards: ASTM D6913-17—Standard Test Methods for Particle-Size Distribution of Soils Using Sieve Analysis [25], and ASTM D4318-17—Standard Test Methods for Liquid Limit, Plastic Limit, and Plasticity Index of Soils [26]. 

#### 2.1.1. Silty Sand

**Figure 1:**
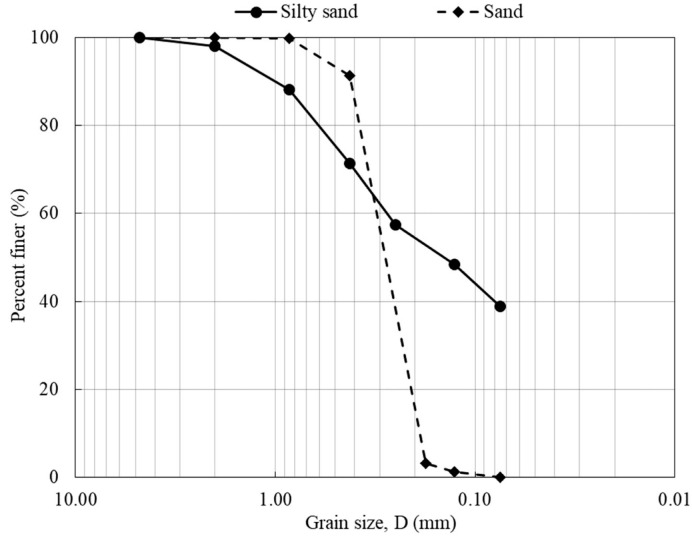
Grain size distribution of the soils used in this study.

From the grain size distribution curve (Figure 1), the concentration of fine particles was 39% with a liquid limit of 49, a plastic limit of 29, and an index of plasticity of 20. According to the Unified Soil Classification System (USCS), the soil is classified as silty sand (SM).

#### 2.1.2. Clean Sand

The sand was characterized by a high percentage of quartz and high uniformity. The coefficient of uniformity and coefficient of curvature were calculated as 1.46, and 0.93, respectively (Figure 1). The percentage of fine particles was below 5%. Therefore, the soil was classified as poorly graded sand (SP), according to USCS.

### 2.2. Biopolymers 

To study the influence of the biopolymer type on the biopolymer-amended soil durability, two types of biopolymers were used in this study: Xantham Gum and Guar Gum. 

#### 2.2.1. Xanthan Gum 

Xanthomonas campestris bacterium creates the biopolymer polysaccharide Xanthan Gum (XG) by inducing the fermentation of a medium containing carbohydrate, such as glucose. In other words, XG is a long-chain polysaccharide having d-glucose, d-mannose, and d-glucuronic acid as building blocks in a molecular ratio of 3:3:2 with a high number of trisaccharide side chains [27]. Dissolving XG in hot or warm water creates non-Newtonian solutions with high pseudoplasticity. XG can be found in the cosmetic and food industry, agriculture, and oil drilling industry [9], and it has been researched for civil engineering purposes [5,6,28,29].

#### 2.2.2. Guar Gum 

Guar Gum (GG) is a galactomannan polysaccharide extracted from Cyamopsis Tetragonolba, known as guar beans or guar. Chemically, a GG biopolymer mainly consists of a high-molecular-weight polysaccharide galactomannan, which is based on a mannan backbone with galactose side groups. The ratio of the two building blocks in a molecular ratio seems to vary slightly depending on the origin of the seed, but the gum is generally considered to contain approximately one galactose building block for every two mannose building blocks [30]. In addition, GG shares certain similarities with XG. For instance, it can be dissolved in hot and cold water, and in the industry is used for similar purposes as XG. It can be found in cosmetic products, food products, oil, and gas drilling industries [30], and it has been researched in civil engineering [6,31,32,33,34].

### 2.3. Specimen Preparation 

The dry base soils were placed in a metal dish and combined with biopolymer powders until uniformly mixed. The biopolymer concentrations used in this study were 0.5, 1, and 2% with respect to the mass of the plain soil. After carefully mixing the dry components (soil and biopolymers), water was added to the mix by spraying and constant stirring. The targeted water content was 16.5% for silty sand and 12% for the clean sand. 

After achieving a uniform mixture, the soil-water-biopolymer mass was placed into molds. Two types of molds (Mold A and Mold B) were used for two different parts of this study. 

Mold A was cylindrical with a diameter of 10.2 cm and a height of 11.6 cm. Silty sand was placed into the Mold A in three lifts and it was compacted with a hammer with a weight of 2.5 kg (Proctor hammer). The same type of mold is typically used for compaction efforts for ASTM D559 and ASTM D698. Each lift was compacted by releasing the hammer 25 times from the height of 30.5 cm. After each lift, the surface was scarified to achieve a better bond within the soil sample. Proctor hammer was omitted for the sand material due to its nature. Sand had a low concentration of fine particles that would have hindered the proper compaction if excessive compaction force was applied. Therefore, sand was carefully tapped into Mold A. Additionally, tapping the sand material into the mold kept the density of the biopolymer-treated sand close to its natural density. Specimens prepared in Mold A were used for durability testing during cyclic wetting and drying. 

Mold B was cubical, with the inner dimensions of 5 cm. Cubical specimens were used for the testing of the compressive strength changes through the wetting-drying cycles. The specimens made out of silty sand were compacted with a metal rod in four lifts. Each lift was pressed 25 times. Sand specimens were gently tapped into the Mold B due to the aforementioned reasons relating to the nature of sand.

**Figure 2:**
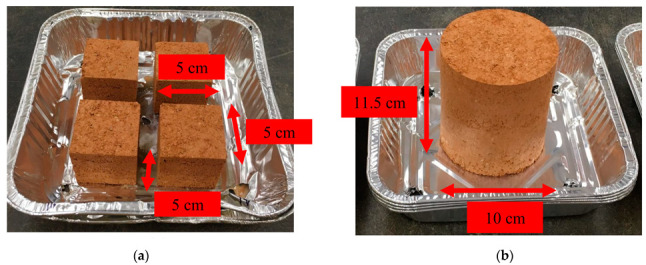
Photos of specimens for (**a**) unconfined compression, and (**b**) durability tests.

All specimens were air-dried in the laboratory at the temperature of 21 °C for five days (cubical specimens, Figure 2a) and seven days (cylindrical specimens, Figure 2b) to increase the biopolymer-soil strength and cure the specimens. 

In addition, specimens made of plain silty sand were prepared in the same manner as the specimens with biopolymer additives. The plain specimens were used for comparison with the biopolymer-treated ones. The samples of the plain sand could not be made because the plain sand used in this research had no cohesion. Therefore, it could not be shaped to the desired dimensions.

### 2.4. Testing 

#### 2.4.1. Durability

The durability testing during cyclic wetting and drying was performed by the guidance of the ASTM D559—Standard Test Methods for Wetting and Drying Compacted Soil-Cement Mixtures [35]. Since ASTM D559 was originally designated for cemented soil, this study introduced certain modifications to the procedure described in ASTM D559. After compaction and air-drying, we measured the mass of specimens and submerged them in water for one hour. The specimens’ mass was measured again after one hour, and specimens were placed in the oven at the temperature of 70 °C for 24 h. After 24 h, samples were taken out of the oven, gently stroked by a brush to remove all loose material, and weighed again before submerging them into the water. The same process was repeated ten times, where one hour in water and 24 h in the oven represents one wetting-drying cycle. This procedure was performed on XG-treated sand, XG-treated silty sand, and GG-treated silty sand. The cylindrical sand specimens with GG degraded after one hour in the water. Therefore, they could not be used for the continuation of the experiment. A similar degradation process happened with the cylindrical specimen of the plain silty sand. They degraded after one hour in the water. Thus, the durability testing of plain silty sand could not be continued. 

#### 2.4.2. Unconfined Compression Test

The unconfined compression test is a widely used test to determine the compressive strength of cohesive materials. The test was performed on the plain and biopolymer-amended silty sand. Moreover, it was performed on the XG-improved sand, whereas untreated sand did not have any cohesion, which was required for this type of test. In addition, sand specimens with GG were not testable for this type of experiment due to their low resistance to water. The unconfined compression test was performed on cubical specimens five days after the preparation and air-drying. Three samples were compressed with an axial strain rate of 1.5%/min, which is in agreement with the ASTM D2166—Standard Test Method for Unconfined Compressive Strength of Cohesive Soil [36]. The remaining specimens were submerged in water at room temperature (21 °C) for 20 min. They were subsequently dried in the oven at 70 °C for 24 h. This process is referred to as one wetting-drying cycle for unconfined compression specimens. Cubical samples of plain silty sand were not tested in the unconfined compression test after wetting and drying due to their degradation in water. After each wetting-drying period, three specimens were tested in the unconfined compression test, while the remaining samples were placed back in the water for 20 min. For the specimens with XG, seven cycles of wetting and drying were carried out through seven days. The unconfined compression test was performed after each cycle except for the fourth and sixth. Most of the cubical specimens with GG were heavily damaged after the first 20 min in water. Therefore, the remaining specimens with GG went through two or three cycles of wetting and drying. 

Furthermore, the healing potential of the XG biopolymer was also investigated by wetting and re-testing previously loaded specimens after their first unconfined compression test. XG-sand cubes were placed in the water for 20 min after they were broken in the unconfined compression test for the first time. They were subsequently placed back in the oven and re-tested for the unconfined compression. The repeated cycle of wetting and drying was conducted to reactivate XG molecules and investigate their regenerative properties on the treated sand. 

## 3. Results and Discussion 

### 3.1. Durability 

**Figure 3:**
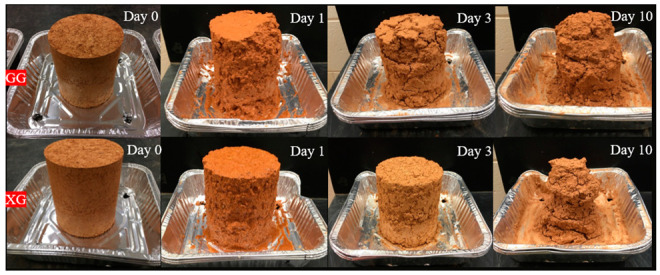
Degradation of silty sand treated with 1% XG (**upper row**) and 1% GG (**bottom row**) due to cyclic wetting and drying.

The durability of the soil was observed through the change of mass through cyclic wetting and drying. The change of mass of the biopolymer-treated soil was calculated for each cycle by the following equation:

% loss = (A/B) × 100 (1)

where *A* is the mass of the soil after each cycle; *B* is the mass of the dry soil after seven days of curing in the air. Several samples were placed in the oven after the preparation and dried for 24 h at 70 °C. Those samples were not tested but they were used for comparison in mass with specimens that were air-dried for 7 days. The differences in mass for the same biopolymer–soil mix were between 2% and 7%. Therefore, we decided that the equation above would be appropriate for the analysis of the durability data. A visual representation of sample degradation through time is shown on biopolymer-treated silty sand in Figure 3.

**Figure 4:**
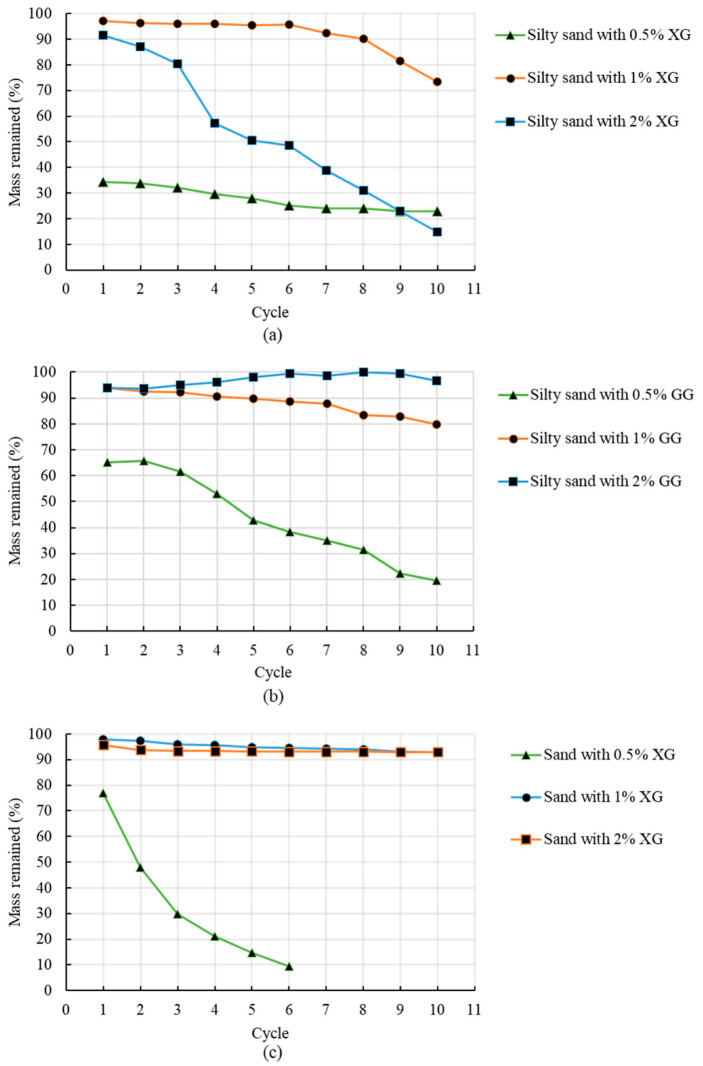
Change in mass for (**a**) Silty sand—XG, (**b**) Silty sand—GG, (**c**) Clean sand—XG.

Figure 4 shows the results of the mass percentages of biopolymer-soil remaining after each cycle. Plain soil samples degraded when submerged under the water for one hour and could not be tested in the designed experiment. The results from Figure 4 indicate that the presence of biopolymers slowed the degradation process for both types of soil. Figure 4 also indicates that the resistance of the soil to cyclic wetting and drying depends on the type of the soil, type of biopolymer additive, and concentration of biopolymer additive. Different biopolymer types, like different soil types, show different reactions with water, which affects the behavior of the soil–biopolymer mixture when exposed to water.

Interestingly, the silty sand with XG showed the best resistance for cyclic wetting and drying at a concentration of 1% XG (Figure 4a). At that concentration, the specimens kept most of the mass up to the sixth wetting-drying cycle. After that, the loss of mass was more noticeable. The same soil type with 0.5% and 2% XG lost more soil mass which indicates that 1% XG could be the optimal water-resistance concentration for this type of soil. There are two reasons behind this: the binding properties of XG and the absorptive properties of the composite material (silty sand and XG). XG is a glue-like binding agent that bridges soil particles. In the mixture with 0.5% XG, soil particles have a weaker biopolymer bond when compared with mixtures with 1 and 2% XG. Therefore, samples with 0.5% XG lost approximately 65% of their initial mass after the first wetting cycle. The plain specimens of silty sand had the weakest particle bond which is the reason they degraded after the first cycle. On the other hand, a question emerges concerning why samples with the highest XG concentration (2%) did not show the best water resistance. The reason for that is the aforementioned absorptive properties of silty sand and XG. The XG attracts and binds water molecules, which further increases the absorbing potential of already swelling plain soil. In other words, the higher presence of XG caused more trapped water. Therefore, to achieve the same water content after each drying for the specimens with 1 and 2% XG, specimens with 2% XG would require a higher drying temperature or longer drying time. The constant higher presence of water can cause the reduction of negative pore pressures that can lower the apparent cohesion and cause the degradation of the soil mass. That resulted in the faster degradation of specimens with 2% XG. However, the loss of the mass under all biopolymer concentrations was significantly reduced when compared with the loss in mass of the plain soil. 

In the case of silty sand amended with GG (Figure 4b), the specimens with higher concentrations have kept more of their soil mass during ten cycles of wetting and drying. When compared with higher concentrations, the samples treated with only 0.5% GG had significantly higher losses of mass between each cycle. Unlike the samples with 1% XG, the samples with 1% GG had a slight gradual loss of mass after each wetting-drying cycle. The samples with 2% GG showed a slight mass increase through the cyclic wetting-drying. That is because of the absorptive properties of silty sand and GG, similar as XG-treated silty sand. The samples with 2% GG lost some amount of soil during wetting-drying, but they also absorbed some water that did not completely evaporate during 24 h in the oven at 70 °C. Therefore, due to the higher absorptive properties of GG at 2% than at 0.5 and 1%, a longer period of drying or a higher drying temperature would be more appropriate for silty sand with 2% GG. Both GG and XG demonstrate a water-absorption nature. However, comparing results of silty sand with 2% GG and 2% XG indicates that GG chains release water molecules somewhat easier than XG chains. Even though XG and GG need to absorb water to activate their bonds with soils, releasing water molecules stiffens the soil–biopolymer bond, which gives GG-treated silty sand an edge over the XG-treated silty sand at a concentration of 2%.

For the clean sand specimens amended with XG (Figure 4c), the loss of mass is relatively low throughout the testing for higher concentrations when compared with silty sand. The reason for that is the fact that the sand has a higher porosity and lower water absorption capacity than silty sand. In other words, water absorption happens only due to the presence of XG. Higher porosity makes the water evaporation relatively faster in the sand than in silty sand. The cementitious effect of hardened XG gave the sand relatively good resistivity to water. However, sand with only 0.5% XG went successfully through only six cycles of wetting and drying before it became wholly degraded.

### 3.2. Unconfined Compression Test 

**Figure 5:**
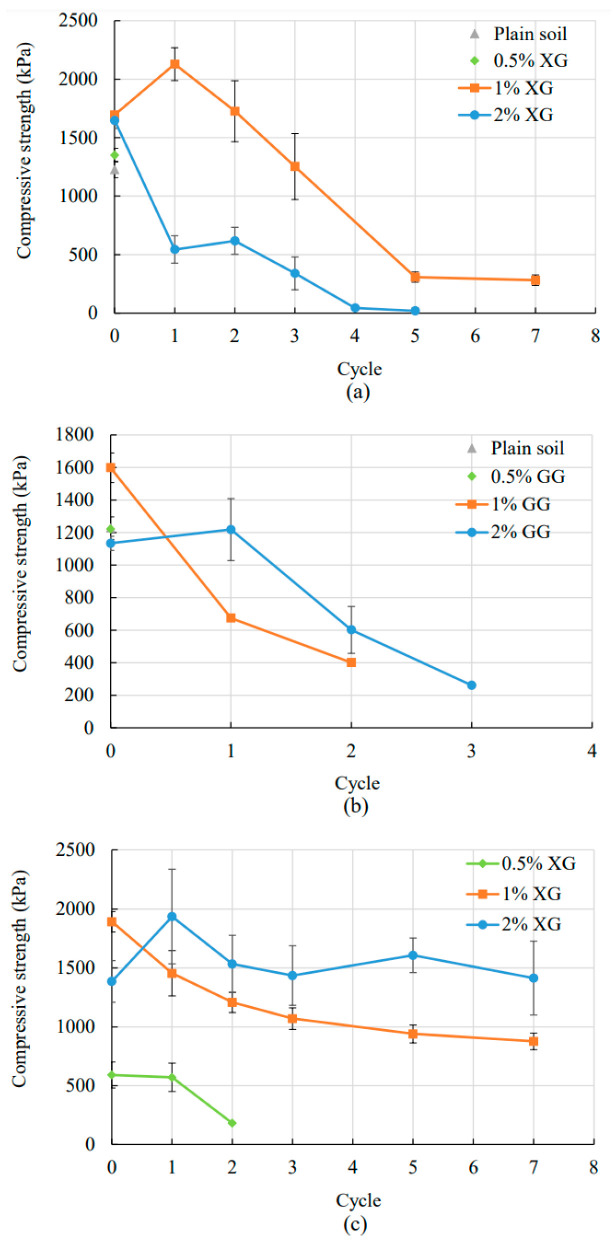
Change in compressive strength for (**a**) Silty sand—XG, (**b**) Silty sand—GG, (**c**) Clean sand—XG.

Figure 5 shows the relationship between the compressive strength and the number of wetting and drying cycles for silty sand (Figure 5a,b) and clean sand (Figure 5c). In all figures, the first point, at cycle zero, represents the compressive strength of specimens tested after five days of air-drying in the laboratory at room temperature. The plain silty sand samples degraded after 20 min in water and could not be tested through cyclic wetting and drying (Figure 5a,b). Plain clean sand samples were not testable because of non-existing cohesion that was needed to fabricate the specimens for this type of testing (Figure 5c). Both types of soil showed fast degradation in water for 0.5% of additives. Therefore, soils amended with 0.5% of biopolymers could not be used for a detailed comparison with soil amended with higher biopolymer concentrations. The exception was the sand treated with 0.5% XG that showed a slightly higher level of water resistance (Figure 5c).

**Figure 6:**
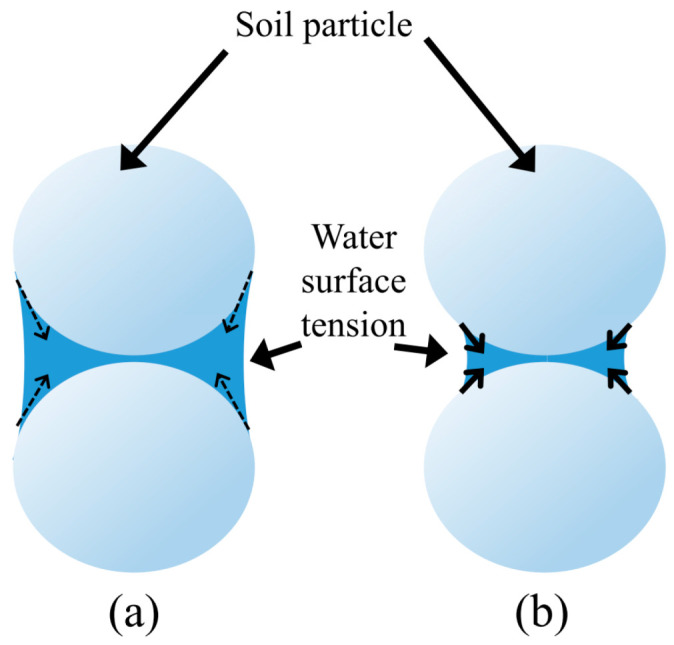
Interaction of water and soil particles: (**a**) a higher degree of saturation—lower surface tension forces, (**b**) lower degree of saturation—higher surface tension forces.

The change in the compressive strength of biopolymer-improved soil with wetting-drying cycles strongly depends on the biopolymer concentration, biopolymer type, and water content. Lower biopolymer concentration in soil results in a reduced number of biopolymer links and subsequent biopolymer–particle bonding. In other words, soils with a lower biopolymer concentration will have smaller compressive strength. On the other hand, a higher percentage of biopolymer causes greater water absorption. The decrease in the water content and degree of saturation increases the surface tension forces between soil particles, which subsequently increases the soil strength (Figure 6). It is noticeable that the biopolymer bond started to weaken after the first wetting and drying cycle because of constant water absorption and the thinning of the biopolymer links. For the specimens made out of the silty sand mixed with 2% XG, the increased biopolymer concentration resulted in higher water absorption than samples with 1% XG. The higher presence of the trapped water caused a more rapid decrease in the compressive strength because of reduced tension forces and loosened biopolymer links.

Figure 5b shows the decrease in the compressive strength for GG-treated silty sand with wetting-drying cycles. The vast majority of the 1% GG-treated cubicles were severely damaged and unusable for the unconfined compression test. Therefore, undischarged specimens went under two cycles of wetting and drying, where the change of their compressive strength was investigated. It is noticeable that after two cycles of wetting and drying, the compressive strength of 1% GG-treated cubicles decreased by 75%. In the case of 1% XG-treated silty sand, the decrease of the compressive strength by 75% would be estimated to happen after the fourth cycle of wetting and drying. The specimens with 2% GG showed better resistivity to water and higher strength through cyclic wetting and drying. The first points, at cycle zero, which represent the specimens after five days of air drying, indicate lower strength for the specimens with 2% GG. This trend was already observed with the samples treated with XG. The reason behind that is that higher concentrations of biopolymer need more air-drying time to completely harden and achieve the maximum strength. The same phenomena happened for the treated sand as well (Figure 5c). 

Figure 5c represents the change of the compressive strength of XG-treated sand with wetting-drying cycles. During seven cycles of wetting and drying, sand with 1% XG kept 46% of the initial strength, while the sand with 2% XG kept 75% of the initial strength. However, the biopolymer-amended sand samples did not sustain their shape and strength at the lowest biopolymer concentration. At the concentration of 0.5% XG, they lost 70% of the initial strength after the second cycle and completely degraded during the third cycle. 

### 3.3. Regenerative Properties of Biopolymers 

**Figure 7:**
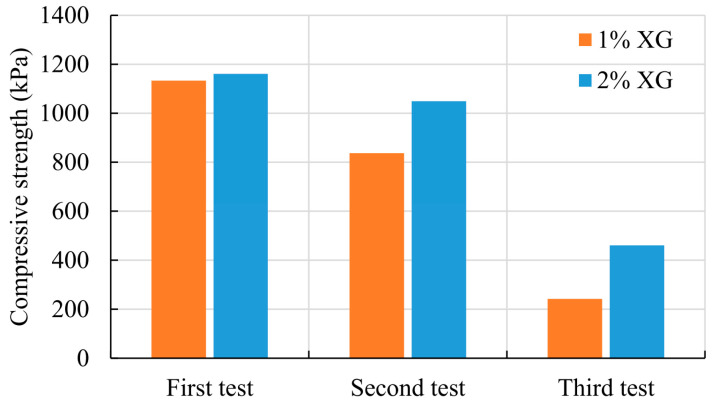
Compressive strength of regenerated sand XG-treated specimens.

Xanthan Gum is one of the partially reversible bond-based biopolymers. That means that it can be brought to the previous state by reapplying the processes that initially induced the change of that state. That reversible nature of XG was examined in biopolymer-treated sand samples that were tested under the unconfined compression test. After the third wetting-drying cycle, that was used to investigate the change in the compressive strength. The broken specimens were used to investigate the healing properties of XG. The broken specimens were submerged for 20 min and dried in the oven for 48 h, as described previously. After that, the same specimens were tested again under the unconfined compression test. The same process was repeated one more time. The results of two XG-treated sand specimens are summarized in Figure 7. The repeated process of wetting-drying stiffened the soil-biopolymer bond, and the XG-treated sand specimen regained some level of the initial strength, which is presented in Figure 7. Higher magnitudes of the compressive strength and the level of the regained strength were achieved for the higher concentration of XG. That is not surprising since a higher concentration of XG causes faster and broader linking of XG molecules with each other and with the surrounding sand particles. 

**Figure 8:**
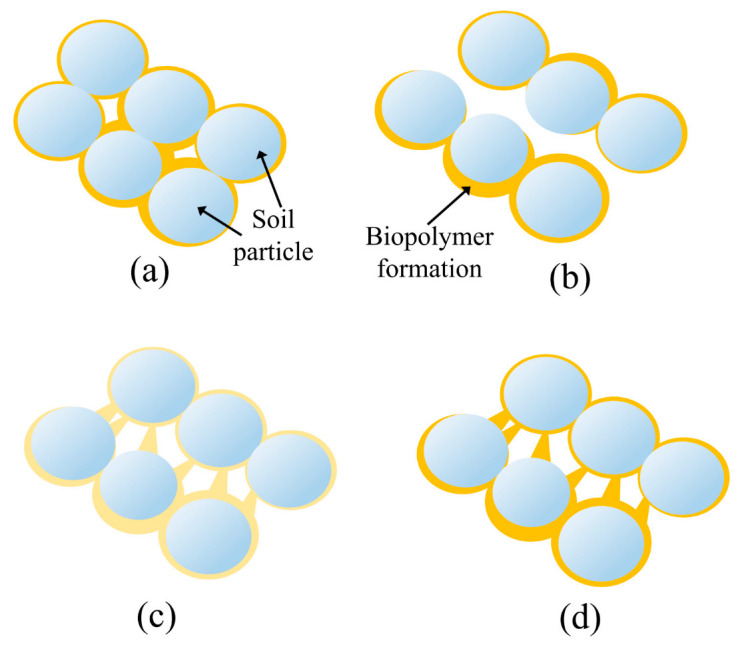
Healing cycle of biopolymer-treated sand: (**a**) sand particles bonded by XG-links, (**b**) breakage of the dry XG-links due to the applied mechanical loading, (**c**) XG- linkages loosen their structure in the contact with water which allows them to interact with the nearby sand particles; and (**d**) sand particles bonded again by new XG-links.

That reversible nature of XG is schematically represented in Figure 8. The sand particles bonded by XG-links (Figure 8a) broke during the unconfined compression test Figure 8b). After the broken specimens of XG-treated sand were put back together and submerged in water, the XG linkages loosened their structure, which allowed them to interact with the nearby sand particles again (Figure 8c) and mend the broken bonds (Figure 8d).

## 4. Conclusions

Recently, biopolymers XG and GG have been shown to be promising environmentally friendly soil stabilization additives. However, they are prone to environmental influence, especially moisture changes. To best to the authors’ knowledge, the previous research studies have not comprehensively investigated the effect of wetting-drying cycles on the strength and mass loss of the different biopolymer-treated soils. Therefore, the main aim of this study is to investigate the effect of wetting-drying cycles on the strength and mass loss of the biopolymer-stabilization. The types of soil used in this study were: silty sand and pure sand. In addition, two types of biopolymers (xanthan gum, and guar gum) and three biopolymer concentrations (0.5%, 1%, 2%) were used as testing variables in this research. 

The first experimental study was focused on observing the change of the mass of the plain and biopolymer-treated soil during cyclic wetting and drying. It was shown that XG reduces the loss of mass for both tested soil types, while GG was only effective when mixed with silty sand. For the silty sand, the most effective concentration of XG to reduce the mass loss during the cyclic wetting and drying process was found to be 1%. The highest used concentration of XG (2%) caused higher entrapment of water, which ultimately led to faster loss of mass. On the other hand, the lowest concentration of XG (0.5%) resulted in too weak biopolymer-soil bonds, which degraded faster. That points to an optimum concentration of XG that works the best with a certain type of soil. For the GG-treated silty sand, the loss of mass was more prominent for lower concentrations. For the XG- treated sand, the loss of mass was relatively low for concentrations of 1% and 2%, which can be explained by higher porosity of sand, which makes water evaporation easier in comparison to the silty sand. A low concentration of 0.5% XG caused weak bonding between soil particles that rapidly degraded. 

The second experiment investigated the change of the compressive strength of biopolymer-treated soil with wetting-drying cycles. XG proved able to reduce the loss of compressive strength in the silty sand and sand, while GG was only mildly effective in the silty sand. However, the increase in the GG concentration reduced strength loss. The concentration of 1% XG was more effective than 2% in reducing the strength loss in the silty sand due to higher water absorption for higher concentrations of XG. The XG-treated sand showed extremely good resistivity to the loss of the compressive strength through cyclic wetting and drying. The higher concentrations of XG resulted in the higher compressive strength of sand. The concentration of 0.5% XG and GG was shown to be mildly or non-effective for the proposed type of testing. 

The broken XG-treated sand specimens were re-submerged, dried, and subsequently tested in the unconfined compression test to study the healing properties of XG-treated sand. It was shown that re-wetting and drying could restore some level of the compressive strength of XG-treated sand. The reason behind it is the regenerative nature of XG, which loosens its structure in water and re-attaches to the nearby soil particles. The healed soil-biopolymer bond stiffens while the sample is subsequently dried and mends the cracks in sandy specimens. Sand samples with higher concentrations of XG were shown to regain more of their lost strength. 

This research study showed that, even though biopolymers tend to be susceptible to water, certain biopolymer types and concentrations can significantly increase the durability of soil to water. It was also shown that the presence of water could activate the regenerative properties of XG, which accentuates its potential for soil stabilization. This research gives a clearer picture of XG and GG utilization, presenting a good option for rapid temporary construction (e.g., embankments) or long-standing construction in areas with an arid climate. The degradation of XG- and GG-treated soil due to longer exposure to water points to a practical way of disposing of the temporary construction elements that are made of the mentioned materials. Due to the non-hazardous nature of these biopolymers, watering and decomposing the XG- and GG-treated soil should not raise environmental concerns. XG and GG also showed favorable characteristics that can be utilized in dust control, erosion, and subgrade stabilization. However, since the water susceptibility of biopolymers is an important factor for their use in industry, this field of research still requires a significant amount of investigation.

## Figures and Tables

**Table 1:** Previous research related to wetting-drying of biopolymer-treated soil.

Authors	Soil	Biopolymer	Testing	Findings
Chen et al. 2015 [22]	Silty Sand	Xanthan Gum Guar Gum	Moisture retention	Moisture retention capacity was higher with the addition of biopolymers.
			Wind Tunnel	- Biopolymer increased the erosion resistance of soil - At higher concentrations, the loss of mass was small during cyclic wetting-drying.
			Penetration test	Increased surface strength with the increase of biopolymer concentration.
Chang et al. 2017 [20]	Poorly Graded Sand	Gellan Gum	Unconfined Compressive Strength Test	- Reduction of the compressive strength was gradual. - The strength remained high after 10 wetting-drying cycles (i.e., >70% of the initial strength).
Chen et al. 2019 [21]	Well Graded Sand	Xanthan Gum	Direct Shear	Reduction of friction angle, cohesion, and peak shear stress was gradual.
Lemboye et al. 2021 [23]	Poorly Graded Sand	Acacia Gum Sodium Alginate Pectin	Wind Tunnel	- Biopolymer increased the erosion resistance of the soil. - Wetting-drying increased the loss of mass.
			Penetration Test	- Biopolymer increased the surface strength of the soil. - Wetting-drying reduced surface strength.
Adamczuk and Jozefaciuk, 2021 [24]	Sand Silt	Chitosan (two types)	Unconfined Compressive Strength Test	Strength changes after wetting-drying depended on soil type, biopolymer type, and biopolymer concentration.

## Data Availability

The data presented in this study are available on request from the corresponding author.

## References

[1] Sanjuán M.Á., Andrade C., Mora P., Zaragoza A. (2020). Carbon dioxide uptake by cement-based materials: A Spanish case study. Appl. Sci..

[2] Chang I., Im J., Cho G.-C. (2016). Introduction of Microbial Biopolymers in Soil Treatment for Future Environmentally-Friendly and Sustainable Geotechnical Engineering. Sustainability.

[3] Umar M., Kassim K.A., Ping Chiet K.T. (2016). Biological process of soil improvement in civil engineering: A review. J. Rock Mech. Geotech. Eng..

[4] Cho G.-C., Chang I. Cementless Soil Stabilizer—Biopolymer. Proceedings of the 2018 World Congress on Advances in Civil, Environmental & Materials Research (ACEM18).

[5] Soldo A., Miletić M. (2019). Study on Shear Strength of Xanthan Gum-Amended Soil. Sustainability.

[6] Soldo A., Miletić M., Auad M.L. (2020). Biopolymers as a sustainable solution for the enhancement of soil mechanical properties. Sci. Rep..

[7] Hou C.T., Barnabe N., Greaney K. (1986). Biodegradation of xanthan by salt-tolerant aerobic microorganisms. J. Ind. Microbiol..

[8] Shahidi F., Synowiecki J. (1991). Isolation and characterization of nutrients and value-added products from snow crab (Chionoecetes opilio) and shrimp (Pandalus borealis) processing discards. J. Agric. Food Chem..

[9] Katzbauer B. (1998). Properties and applications of xanthan gum. Polym. Degrad. Stab..

[10] Bourriot S., Garnier C., Doublier J.-L. (1999). Phase separation, rheology and microstructure of micellar casein–guar gum mixtures. Food Hydrocoll..

[11] Volman J.J., Ramakers J.D., Plat J. (2008). Dietary modulation of immune function by βglucans. Physiol. Behav..

[12] Chen R., Zhang L., Budhu M. (2013). Biopolymer Stabilization of Mine Tailings. J. Geotech. Geoenviron. Eng..

[13] Latifi N., Horpibulsuk S., Meehan C.L., Abd Majid M.Z., Tahir M.M., Mohamad E.T. (2017). Improvement of Problematic Soils with Biopolymer—An Environmentally Friendly Soil Stabilizer. J. Mater. Civ. Eng..

[14] Wiszniewski M., Skutnik Z., Biliniak M., Çabalar A.F. (2017). Some geomechanical properties of a biopolymer treated medium sand. Annals of Warsaw University of Life Sciences—SGGW. Land Reclam..

[15] Hataf N., Ghadir P., Ranjbar N. (2018). Investigation of soil stabilization using chitosan biopolymer. J. Clean. Prod..

[16] Ayeldeen M., Negm A., El-Sawwaf M., Kitazume M. (2017). Enhancing mechanical behaviors of collapsible soil using two biopolymers. J. Rock Mech. Geotech. Eng..

[17] Orts W.J., Sojka R.E., Glenn G.M. (2000). Biopolymer additives to reduce erosion-induced soil losses during irrigation. Ind. Crops Prod..

[18] Kavazanjian E.J., Iglesias E., Karatas I. (2009). Biopolymer soil stabilization for wind erosion control. Proceedings of the 17th International Conference on Soil Mechanics and Geotechnical Engineering.

[19] Chang I., Prasidhi A.K., Im J., Cho G.-C. (2015). Soil strengthening using thermo-gelation biopolymers. Constr. Build. Mater..

[20] Chang I., Im J., Lee S.-W., Cho G.-C. (2017). Strength durability of gellan gum biopolymer treated Korean sand with cyclic wetting and drying. Constr. Build. Mater..

[21] Chen C., Wu L., Harbottle M. (2020). Exploring the effect of biopolymers in near-surface soils using xanthan gum—Modified sand under shear. Can. Geotech. J..

[22] Chen R., Lee I., Zhang L. (2015). Biopolymer stabilization of mine tailings for dust control. J. Geotech. Geoenvironmental Eng..

[23] Lemboye K., Almajed A., Alnuaim A., Arab M., Alshibli K. (2021). Improving sand wind erosion resistance using renewable agriculturally derived biopolymers. Aeolian Res..

[24] Adamczuk A., Jozefaciuk G. (2022). Impact of Chitosan on the Mechanical Stability of Soils. Molecules.

[25] (2017). Standard Test Methods for Particle-Size Distribution (Gradation) of Soils Using Sieve Analysis.

[26] (2017). Standard Test Methods for Liquid Limit, Plastic Limit, and Plasticity Index of Soils.

[27] Jindal N., Singh Khattar J., Grumezescu A.M., Holban A.M. (2018). Chapter 4—Microbial Polysaccharides in Food Industry. Biopolymers for Food Design, Handbook of Food Bioengineering.

[28] Wiszniewski M., Skutnik Z., Cabalar A.F. (2013). Laboratory assessment of permeability of sand and biopolymer mixtures. Annals of Warsaw University of Life Sciences—SGGW. Land Reclam..

[29] Chang I., Im J., Prasidhi A.K., Cho G.-C. (2015). Effects of Xanthan gum biopolymer on soil strengthening. Constr. Build. Mater..

[30] Rayment P., Ellis P.R., Caballero B. (2003). GUMS|Nutritional Role of Guar Gum. Encyclopedia of Food Sciences and Nutrition.

[31] Thombare N., Jha U., Mishra S., Siddiqui M.Z. (2016). Guar gum as a promising starting material for diverse applications: A review. Int. J. Biol. Macromol..

[32] Ayeldeen M.K., Negm A.M., El Sawwaf M.A. (2016). Evaluating the physical characteristics of biopolymer/soil mixtures. Arab. J. Geosci..

[33] Dehghan H., Tabarsa A., Latifi N., Bagheri Y. (2018). Use of xanthan and guar gums in soil strengthening. Clean Technol. Environ. Policy.

[34] Toufigh V., Kianfar E. (2019). The effects of stabilizers on the thermal and the mechanical properties of rammed earth at various humidities and their environmental impacts. Constr. Build. Mater..

[35] (2015). Standard Test Methods for Wetting and Drying Compacted Soil-Cement Mixtures.

[36] (2016). Standard Test Method for Unconfined Compressive Strength of Cohesive Soil.

